# Traditional ecological knowledge and mental health education for sustainable community well-being

**DOI:** 10.3389/fpsyg.2026.1888222

**Published:** 2026-06-30

**Authors:** Shuai Xu, Ke Xu, Waqar Ahmad, Valentin-Marian Antohi, Costinela Fortea, Mihaela Neculita

**Affiliations:** 1 School of Marxism, Sias University, Zhengzhou, China; 2 School of Economics and Management, Jiangxi Normal University, Nanchang, Jiangxi, China; 3 School of Economics, International Islamic University, Islamabad, Pakistan; 4 Department of Business Administration, Dunarea de Jos University of Galați, Galați, Romania; 5 Department of Finance, Accounting, and Economic Theory, Transilvania University of Brașov, Brașov, Romania; 6 Department of Economics, Dunarea de Jos University of Galati, Galați, Romania

**Keywords:** community well-being, indigenous knowledge, mental health education, sustainable development, traditional ecological knowledge

## Abstract

Traditional ecological knowledge (TEK) refers to the traditional and cumulative knowledge, skills, and beliefs about the relationship between humans and nature that are passed on from generation to generation within indigenous cultures. As communities worldwide grapple with mental health issues, environmental deterioration, social disintegration, and the loss of indigenous knowledge systems, there is a growing interest in approaches that integrate TEK to promote holistic well-being. The current mixed-methods study was carried out in Southwestern China among ethnic communities to investigate the role of TEK in mental health education and sustainable community well-being. Data were collected through a validated 32-item questionnaire (internal consistency reliability: Cronbach’s *α* = 0.89) completed by 240 participants and semi-structured interviews, focus group discussions, and participatory observations of 48 participants. Pearson correlation analysis revealed that TEK participation was positively associated with mental health awareness (r = 0.71, *p* < 0.01), emotional resilience (r = 0.78, *p* < 0.01), and sustainability engagement (r = 0.74, *p* < 0.01). Multiple linear regression analysis also revealed that TEK participation was the most significant predictor of emotional resilience (*β* = 0.48, *p* < 0.001), followed by mental health awareness (β = 0.31, *p* < 0.001) and sustainability engagement (β = 0.24, *p* < 0.001), accounting for 64% of the variance in emotional resilience (R^2^ = 0.64). Results showed that communities with higher levels of TEK participation exhibited stronger social cohesion, lower perceived stress, higher emotional resilience, and higher environmental responsibility.

## 1 Introduction

Traditional ecological knowledge (TEK) refers to the accumulated body of environmental observations, agricultural traditions, medicinal practices, seasonal forecasting, biodiversity conservation techniques and spiritual understandings of nature that indigenous and local communities have developed over generations through their interactions with ecosystems (Mekonen, 2017), which are distinct from modern scientific systems in being more connected between humans and ecosystems (Dinker, 2024). The importance of TEK for sustainability, resilience and holistic health is now receiving increased global attention (Galvin, 2020).

Mental health has become one of the most urgent global public health issues of the twenty-first century, with rapid urbanization, climate change, social isolation, economic inequalities and technological transformations all leading to increased levels of stress, anxiety, depression and emotional instability among communities (Ríos-Rodríguez et al., 2024). In environmental psychology, public health and sustainability studies, the relationship between ecological systems and psychological well-being is becoming increasingly important, as research shows that exposure to natural environments can enhance emotional regulation, reduce stress, build social relationships and improve cognitive functioning (Galvin, 2020). Communities with a strong ecological tradition also tend to have collective support systems, cultural rituals and shared identities that bolster resilience in the face of social or environmental shocks (Galvin, 2020). The cultural continuity, environmental awareness and emotional well-being aspects of TEK may offer useful frameworks for mental health education.

Indigenous peoples around the world have used storytelling, ceremonies, communal farming, medicinal plants and land-based learning to keep social harmony and psychological balance (Eagle, 2025), which preserve ecological resources and contribute to emotional healing, identity formation and intergenerational bonding. Traditional healing systems may view mental well-being as intertwined with social relationships, spiritual practices and environmental conditions (Marques et al., 2021), which is very different from highly individualized approaches often seen in modern mental health systems.

The erosion of traditional ecological knowledge is occurring in many parts of the world as a result of globalization and modernization, as younger generations become more disconnected from indigenous languages, cultural practices and environmental traditions through urban migration, industrialization and changes in educational systems (Aswani et al., 2018). The loss of traditional ecological knowledge has implications for biodiversity conservation, but also for cultural identity and mental health; the loss of traditional support structures can lead to social alienation, emotional distress, and decreased community cohesion.

Mental health education is designed to increase psychological literacy, emotional awareness, coping mechanisms, and social support within communities, and it should be culturally relevant, socially inclusive, and environmentally responsive (Gueldner et al., 2020). The incorporation of traditional ecological knowledge into mental health education may enhance the educational relevance by linking psychological wellbeing with cultural practices and environmental realities (Finn et al., 2017). Participatory educational experiences through land-based learning activities, ecological storytelling, medicinal plant education, and cultural ceremonies can strengthen environmental awareness and emotional resilience.

Community well-being goes beyond economic development or healthcare access; it also encompasses environmental sustainability, social equity, cultural continuity, educational inclusion and collective resilience (Kimmerer, 2012). Communities that are able to sustain healthy ecosystems, social relationships and cultural identities are more able to cope with environmental challenges and mental health crises; this integration of TEK and mental health education may help to achieve larger sustainability goals through community empowerment and resilience.

Indigenous knowledge systems are becoming increasingly integrated into the work of international organizations and sustainability frameworks, with sustainable development policies emphasizing community participation, cultural preservation, and environmental stewardship (Kohsaka and Rogel, 2021). Many educational and healthcare systems do not fully integrate traditional ecological knowledge into their mental health education, and more interdisciplinary research is needed to understand how ecological traditions can contribute to mental health outcomes and sustainable development at the same time.

This research explores the contribution of TEK to mental health education and to sustainable community well-being, including emotional resilience, community cohesion, psychological awareness, and environmental responsibility, as well as the educational strategies to effectively integrate indigenous ecological knowledge into mental health programs, informed by community experiences, and providing recommendations for policymakers, educators, and healthcare professionals interested in culturally grounded and sustainability-oriented mental health initiatives. The intersection of this interdisciplinary research connects environmental studies, mental health education, sustainability science and indigenous knowledge research and may lead to the development of culturally responsive educational models that promote psychological health and ecological sustainability; ultimately, it represents an approach for integrating traditional ecological knowledge into mental health education for the purpose of enhancing sustainable community well-being in social-ecological contexts.

## 2 Methods

### 2.1 Research design

A mixed-methods research design is applied in this study because the use of a mixed method approach permits the inclusion of both quantitative aspects (community participation, awareness about mental health problems, emotional resilience) and qualitative aspects (ecological practices related to cultural experiences that influence psychological outcomes such as sense of wellbeing and happiness) to better understand the community experience (Doucerain et al., 2016).

The study was carried out in a rural and semi-rural context, through an established community-based participatory research framework, which included elders, educators, youth representatives, and local practitioners, with ethics approval prior to conducting the study and informed consent obtained from the participants (Branquinho et al., 2020). The study was completed over the course of a year in communities that still engage in traditional ecological practices and involved multiple phases of data collection, including preliminary consultations, field observations, educational assessments, and thematic interviews. The mixed-methods design allowed for triangulation of findings (to increase reliability and validity) and included statistical trends alongside narrative experiences that together yielded a more comprehensive understanding of how ecological traditions provide mental health education and community resilience.

### 2.2 Study area and participant selection

The study was conducted in selected rural and ethnic communities located in the Southwest China and Jiangxi provinces, China where the traditional ecological knowledge (TEK) remains actively practiced through medicinal plant use, community agriculture, forest stewardship, seasonal ceremonies, water conservation practices, and land-based cultural traditions. The participating communities included Yi, Miao (Hmong), Dong, and Yao populations, as well as traditional rural agricultural communities that maintain strong connections with local ecosystems and cultural heritage. These communities were selected because of their continued engagement with the ecological traditions and their relevance for examining the relationship between TEK, mental health education and the sustainable community well-being.

Purposive sampling was employed to recruit the participants possessing direct knowledge of the ecological traditions and community educational practices (Okafor et al., 2022). Participants included community elders, traditional healers, school teachers, healthcare workers, youth leaders, environmental practitioners and the local administrative representatives. A total of 240 individuals participated in the quantitative survey, while 48 participants were involved in the semi-structured interviews and focus group discussions. Inclusion criteria required participants to have resided within the community for at least five years and to possess familiarity with local ecological practices. Efforts were made to ensure representation across gender, age groups, educational backgrounds and occupational categories to capture diverse perspectives regarding ecological knowledge transmission, mental health education and community sustainability.

### 2.3 Data collection procedures

To investigate the impact of traditional ecological knowledge, mental health education, and community well-being, multiple complementary methods were used to collect data (Duarte et al., 2019). A 32-item structured questionnaire was developed based on a literature review of traditional ecological knowledge, mental health promotion, emotional resilience, and sustainability education (Duarte et al., 2019). The questionnaire comprised four domains, with eight items per domain: Traditional Ecological Knowledge Participation, Mental Health Awareness, Emotional Resilience, and Sustainability Engagement, and responses were measured using a five-point Likert scale ranging from 1 (strongly disagree) to 5 (strongly agree).

Content validity was determined through expert review by environmental psychology specialists, indigenous studies specialists, and community mental health specialists prior to implementation, and a pilot assessment was conducted with participants from surrounding communities to assess clarity, cultural appropriateness, and comprehensibility of the instrument. Results of reliability analysis showed high internal consistency with Cronbach’s alpha values of 0.88 for TEK Participation, 0.86 for Mental Health Awareness, 0.91 for Emotional Resilience, 0.84 for Sustainability Engagement, and a total Cronbach’s alpha value of 0.89. Interviews with elders, traditional healers, educators, healthcare practitioners, and community leaders and focus group discussions with youth, women, and community leaders were conducted to explore ecological knowledge transmission, storytelling traditions, medicinal plant practices, environmental stewardship, cultural identity, emotional resilience, and perceptions of community mental health challenges. Field observations were made during community gatherings, environmental ceremonies, agricultural activities, ecological conservation initiatives, educational workshops, and cultural celebrations, and detailed field notes, observation records, and reflective journals were kept. This combination of questionnaire surveys, interviews, focus groups, and participant observation allowed triangulation across data sources and increased the credibility of the findings (Jidong et al., 2021).

### 2.4 Integration of the traditional ecological knowledge in mental health education

The methodology considered the integration of traditional ecological knowledge into community mental health education programs, including formal and informal educational activities incorporating ecological practices, cultural teachings and environmental values, such as storytelling sessions, medicinal plant workshops, nature-based counseling practices, community farming programs, and seasonal ecological celebrations (Datta, 2018). Researchers evaluated educational programs based on participant engagement, cultural relevance, emotional learning outcomes, and sustainability awareness, the involvement of elders and traditional knowledge holders in educational processes, and how elders can serve as mentors sharing ecological knowledge, moral values, and coping strategies with the next generation, and how their involvement supports intergenerational learning and cultural identity (Atkins et al., 2016). Reviewing mental health education materials, interviews with educational facilitators, and analysis of how ecological traditions were incorporated into mental health education helped determine how culturally grounded educational approaches can contribute to emotional resilience, social inclusion, and environmental responsibility, and how ecological traditions can be incorporated into mental health education as a holistic approach to community development.

### 2.5 Data analysis techniques

Analyzing the research results, the researchers applied complementary analytical techniques, including descriptive and inferential statistical analysis of both quantitative and qualitative data (Alem, 2020), survey responses were coded and entered into statistical software for frequencies, percentages, mean scores, and correlation analyses to assess the relationship between ecological participation, mental health awareness, emotional resilience, and community engagement, while the qualitative data from the interviews, focus groups, and field observations were analyzed through thematic analysis with audio recordings transcribed, translated where necessary, and systematically coded based on emerging themes, and researchers observed recurring patterns in cultural identity, emotional support, environmental stewardship, educational participation, and sustainability practices. Pearson correlation analysis was used to explore the relationships between TEK participation, mental health awareness, emotional resilience, and sustainability engagement. Then, multiple linear regression analysis was conducted to determine factors that predict emotional resilience, and regression coefficients (*β*), standard errors, t-values, *p*-values, model fit statistics (R^2^ and adjusted R^2^), and confidence indicators were calculated and reported. A *p*-value of less than 0.05 was considered statistically significant.

By triangulation, researchers compared findings across different data sources and participant groups (Moon, 2019) to enhance the credibility and validity of their interpretations, and they conducted member-checking sessions (selected participants reviewed preliminary findings to confirm cultural accuracy and contextual relevance). Narrative analysis was utilized to explore how participants narrated their experiences of the ecological traditions and psychological well-being (Ruini et al., 2017), local metaphors, cultural symbols, and storytelling patterns were examined that reflected the community perspective on mental health and sustainability. The findings of the study, combining statistical and thematic analysis, identified measurable trends and culturally embedded experiences, and the integration of statistical and thematic findings enhanced the overall interpretation of how traditional ecological knowledge impacted mental health education and sustainable community well-being.

### 2.6 Ethical considerations and research limitations

As indigenous knowledge systems and mental health discussions carry significant cultural implications, ethical considerations were central to the research process, and participants were provided with prior informed consent regarding the aims of the study, their right to withdraw at any time, their confidentiality, and how the results of the research would be utilized (Petrova et al., 2016). Cultural protocols related to traditional ecological knowledge were honored, community leaders and elders were consulted, and culturally restricted details of certain ceremonies or spirits were omitted from public documentation.

Confidentiality was ensured by assigning participant identifiers that were not linked to their names and by following strict protocols for storing data. Researchers refrained from language that might pathologize mental health experiences or mischaracterize indigenous traditions. Findings were presented to community members in feedback sessions to allow for interpretation of the results together. Even with detailed methodological planning, several limitations were noted (Lewis et al., 2015), including a focus on specific communities that may not reflect the breadth of ecological traditions within other cultural contexts, challenges with language translation that may have affected the interpretation of some narratives and symbolic meanings, and the cross-sectional nature of the research that made it difficult to assess the long-term effects of the ecological education on mental health outcomes. This study offers important interdisciplinary insight into the relationships between traditional ecological knowledge, mental health education, and sustainable community well-being and serves as a call for additional longitudinal and comparative studies to further advance this new field.

## 3 Results

### 3.1 Community participation in traditional ecological practices

Results showed that nearly 78% of the survey participants across the study areas were engaged in some form of ecological activities such as medicinal plant collection, community farming, water conservation, forest stewardship and seasonal cultural ceremonies on a regular basis. Elder participants were the most involved in ecological activities, and younger participants were moderately involved through educational and community programs. Strength of 240 participants completed the quantitative survey, while 48 participants participated in interviews and focus group discussions. The survey demonstrated excellent internal consistency, with an overall Cronbach’s alpha coefficient of 0.89, indicating the high reliability across the measured constructs.

The demographic distribution of the study participants by gender, age group, occupation, educational background, and their level of participation in traditional ecological activities, which field observations showed were often social gatherings that reinforced interpersonal relationships and a sense of group identity, providing emotional support, intergenerational interaction and collaborative problem solving that many respondents viewed as necessary for a sense of belonging and cultural continuity, are shown in Table 1.

**Table 1:** Sociodemographic profile of the study participants.

Variable	Category	Frequency (*n*)	Percentage (%)
Gender	Male	118	49.2
	Female	122	50.8
Age group	18–30 years	62	25.8
	31–45 years	88	36.7
	46–60 years	56	23.3
	Above 60 years	34	14.2
Occupation	Farmers	72	30.0
	Teachers	34	14.2
	Traditional healers	18	7.5
	Healthcare workers	26	10.8
	Environmental practitioners	30	12.5
	Community leaders	22	9.2
	Youth representatives	38	15.8
Educational status	Primary education	54	22.5
	Secondary education	96	40.0
	Higher education	90	37.5
Ecological participation	High participation	112	46.7
	Moderate participation	86	35.8
	Low participation	42	17.5

Table 2 summarizes the traditional ecological practices identified in the study and their contributions to mental health promotion, community resilience and environmental sustainability. Participants in focus group discussions reported that ecological practices helped them to be emotionally stable and reduce stress, and that time spent in nature was often described as peaceful, relaxing and spiritual, but younger participants said modernization and urban migration reduced their involvement in traditional ecological activities, and limited access to traditional lands, as well as changing educational priorities and digital lifestyles, were barriers to participation. Nonetheless, communities with active ecological programs were observed to have higher levels of collective resilience and social cohesion.

**Table 2:** Traditional ecological practices and their contributions to community well-being.

Traditional ecological practice	Community function	Mental health contribution	Sustainability benefit
Medicinal plant use	Traditional healing	Stress reduction and emotional comfort	Biodiversity conservation
Community farming	Collective food production	Social bonding and emotional support	Sustainable agriculture
Storytelling traditions	Cultural education	Identity formation and resilience	Preservation of the cultural heritage
Seasonal Rituals	Community celebration	Emotional stability and belonging	Ecological awareness
Water conservation practices	Resource management	Reduced environmental anxiety	Water sustainability
Forest stewardship	Ecosystem protection	Nature connectedness and relaxation	Forest conservation
Nature-based counseling	Informal emotional support	Improved coping strategies	Environmental stewardship
Intergenerational learning	Knowledge transfer	Strengthened social cohesion	Cultural continuity

**Figure 1:**
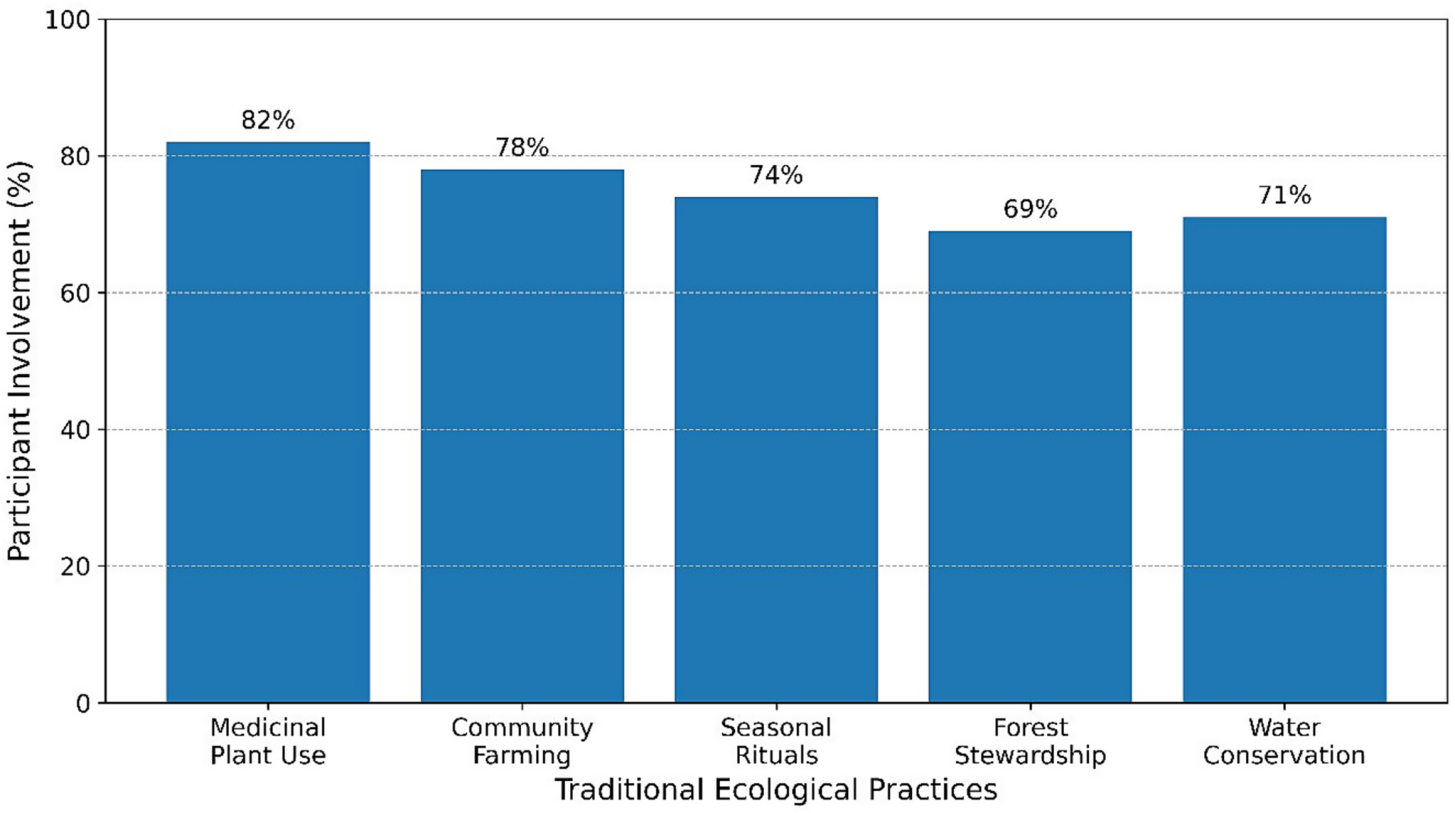
Community participation in traditional ecological practices across study areas.

Figure 1 illustrates the percentage distribution of participant involvement in the major traditional ecological practices including medicinal plant use, community farming, seasonal rituals, forest stewardship and the water conservation activities across selected communities.

**Figure 2:**
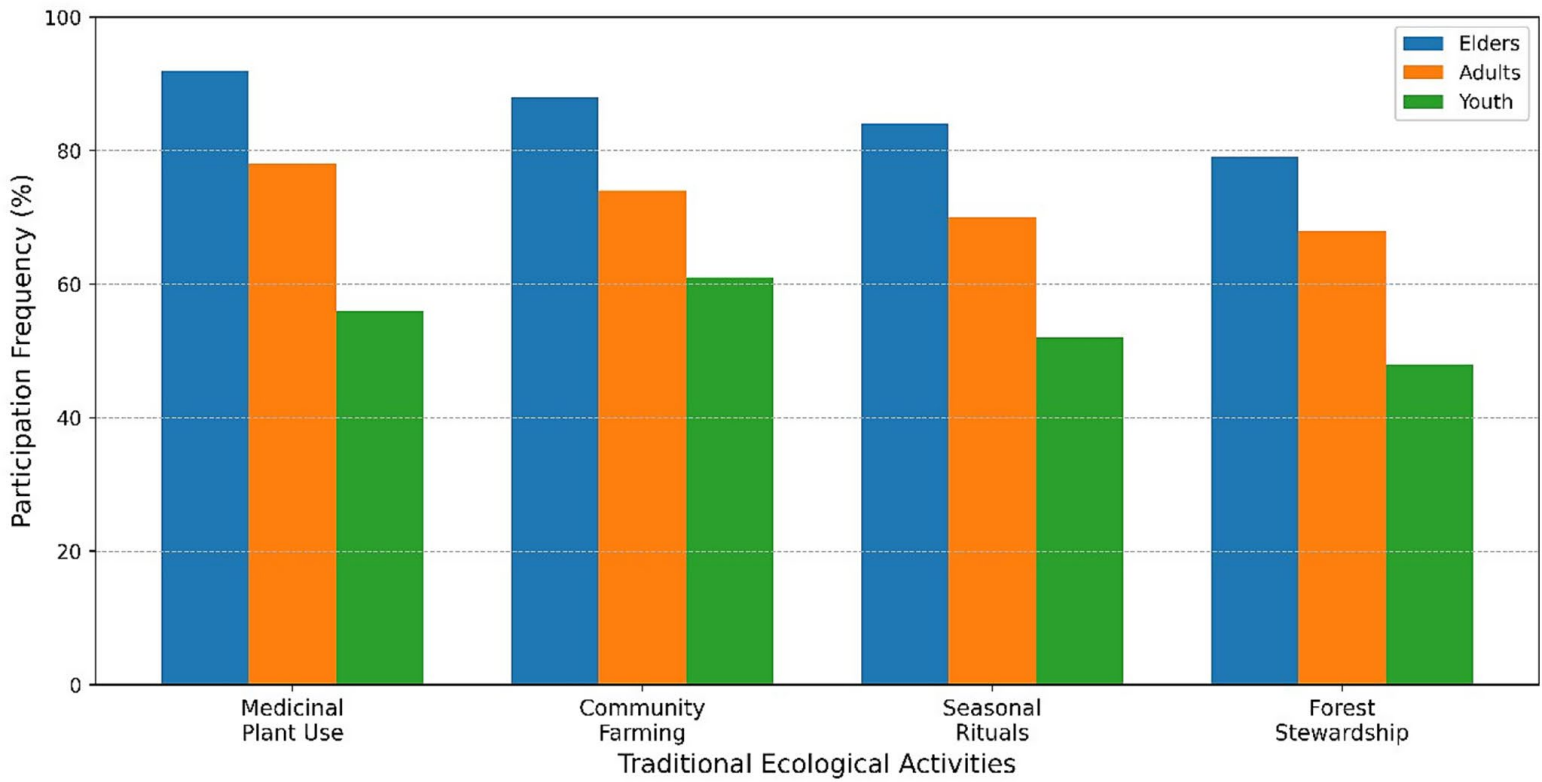
Distribution of the traditional ecological activities among community participants.

The relative frequency of ecological participation of elders, adults, and youth participants, illustrating generational differences in participation with traditional ecological knowledge systems, is shown in Figure 2, while traditional ecological practices contribute to environmental sustainability, emotional well-being, and connectedness to the community (Figures 1, 2; Tables 1, 2) and should be protected as a part of a comprehensive mental health and sustainability strategy.

### 3.2 Influence of the ecological knowledge on emotional resilience

Traditional ecological knowledge was significantly correlated with emotional resilience of community members: survey data suggested that participants who were more involved in ecological traditions reported lower levels of the stress and higher levels of the emotional stability than participants with less involvement in ecological traditions; interview data suggested that ecological knowledge provided coping mechanisms during social or environmental uncertainty: traditional practices such as storytelling, ceremonial gatherings, and medicinal plant use were often reported as sources of comfort and emotional guidance; cultural teachings of patience, collective responsibility, gratitude toward nature, and spiritual balance were reported as leading to psychological resilience.

Table 3 compares indicators of emotional resilience, stress reduction, social connectedness and cultural identity across different levels of participation in the practice of traditional ecological knowledge. Elders, who transmitted emotional coping strategies through oral traditions and land-based experiences, shared that younger participants who interacted with them had improved self-confidence, stronger cultural identity, and greater emotional awareness. A number of participants also said that learning ecological traditions made them feel more connected to their heritage and purpose. Nature-based activities such as forest walks, agricultural work and river conservation programs also created peaceful settings for contemplation and conversation, reducing interpersonal tensions and strengthening cooperative relationships in communities. Pearson correlation analysis demonstrated the significant positive relationships among all the study variables. Multiple linear regression analysis demonstrated that the overall model was statistically significant (*F* = 89.70, *p* < 0.001) and explained 64% of the variance in emotional resilience (R^2^ = 0.64; Adjusted R^2^ = 0.63). TEK participation emerged as the strongest predictor of emotional resilience (*β* = 0.48, SE = 0.05, 95% CI = 0.38–0.58, *p* < 0.001), followed by mental health awareness (β = 0.31, SE = 0.06, 95% CI = 0.21–0.41, *p* < 0.001) and sustainability engagement (β = 0.24, SE = 0.05, 95% CI = 0.15–0.34, *p* < 0.001). These findings indicate that higher engagement with traditional ecological knowledge and related community-based educational practices was positively associated with greater emotional resilience among participants.

**Table 3:** Comparative mental health outcomes across levels of ecological participation.

Mental health indicator	High TEK participation	Moderate TEK participation	Low TEK participation
Emotional resilience score	8.8 ± 0.6	7.1 ± 0.8	5.9 ± 1.1
Stress reduction level	82%	64%	38%
Social connectedness	89%	70%	44%
Community engagement	91%	68%	40%
Sense of cultural identity	94%	72%	46%
Anxiety reduction	78%	57%	32%

**Figure 3:**
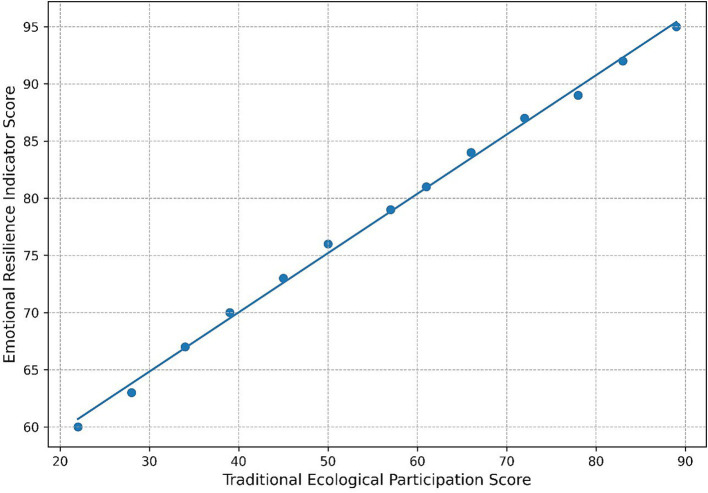
Association between traditional ecological participation and emotional resilience indicators.

A positive regression relationship between traditional ecological participation scores and emotional resilience indicators showed that higher ecological participation scores were associated with higher emotional resilience scores, suggesting that higher ecological participation scores were related to higher scores on the emotional resilience indicators (Figure 3). A few respondents expressed concern over the loss of ecological knowledge over time, and the erosion of traditional practices was linked to increased social fragmentation and reduced community support systems. This suggests that the emotional resilience afforded by traditional ecological knowledge is linked to cultural identity, social support, and meaningful interaction with the natural world (Figure 3; Table 3).

### 3.3 Mental health awareness through community education programs

The study found that communities that incorporated ecological traditions into educational programs had significantly better mental health awareness, with more participants participating in discussions around mental health issues, with 71% of participants in culturally grounded educational programs knowing more about stress management techniques, emotional communication and community support resources. Participants said that the ecological education programs made mental health topics more relevant and less stigmatized because they were linked to cultural practices that they knew. Teachers and community facilitators said that including traditional ecological knowledge in educational activities enhanced participation and fostered intergenerational communication, especially with experiential learning approaches such as gardening projects, forest-based learning and cultural storytelling sessions that promoted self-expression, teamwork and emotional reflection.

Table 4 outlines community-based educational activities integrating traditional ecological knowledge with mental health education and their associated psychological and sustainability outcomes.

**Table 4:** Community-based educational approaches linking ecological knowledge and mental health.

Educational activity	Target group	Psychological outcome	Sustainability outcome
Storytelling workshops	Youth and children	Improved emotional expression	Cultural preservation
Medicinal plant education	Adults and elders	Increased stress management awareness	Biodiversity protection
Community gardening	Families	Enhanced cooperation and belonging	Sustainable food systems
Forest-based learning	Students	Improved concentration and resilience	Environmental stewardship
Ritual and ceremony participation	Community members	Emotional stability and identity	Cultural continuity
Nature-based counseling	Vulnerable Groups	Reduced anxiety and isolation	Ecological awareness

**Figure 4:**
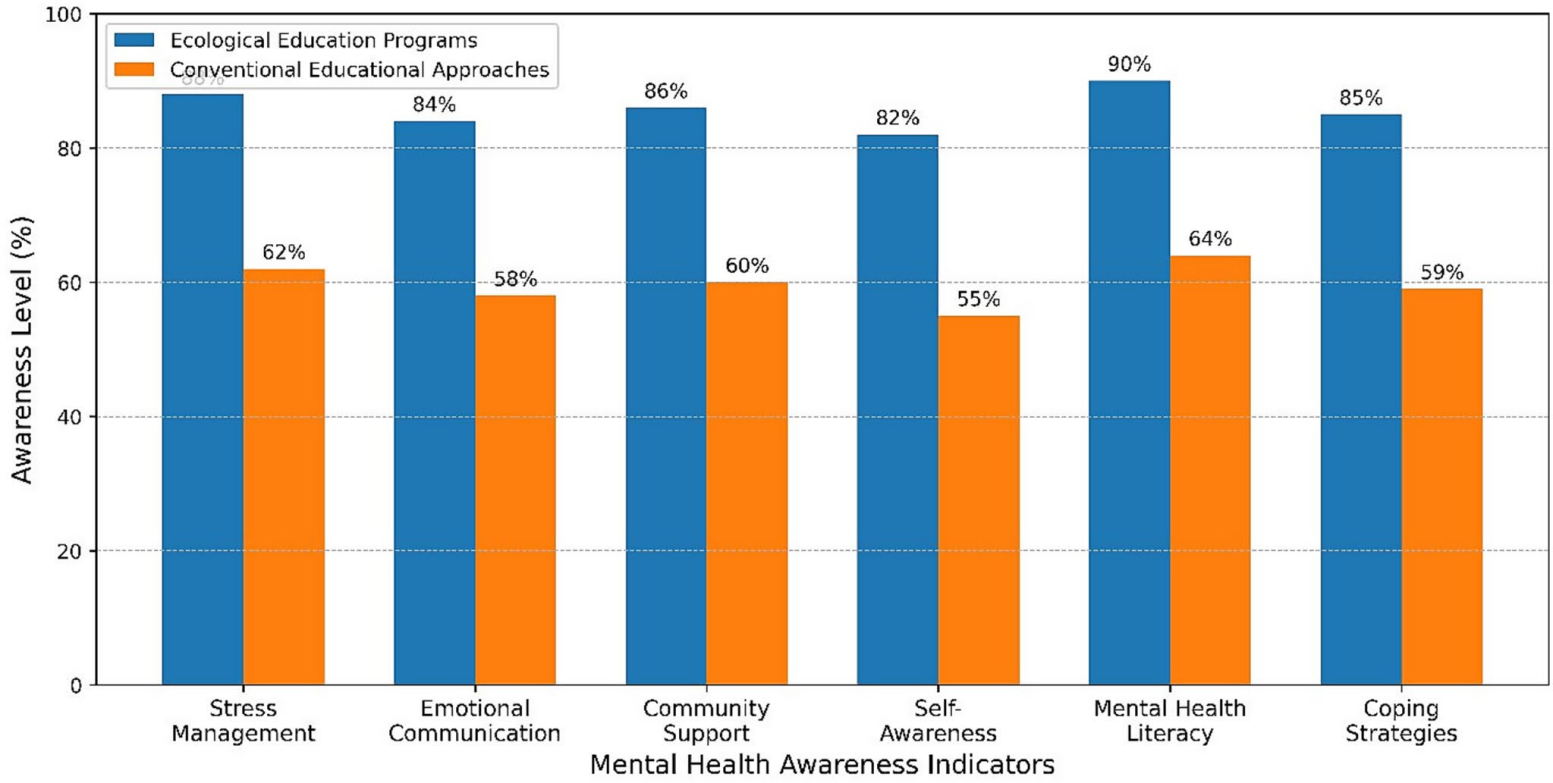
Mental health awareness levels in ecological education programs.

Figure 4 shows the comparison of mental health awareness levels between participants involved in ecological education programs and participants who were exposed only to traditional educational methods. The focus group discussions revealed that ecological education increased knowledge of the environmental responsibility and sustainability, with participants realizing that healthy ecosystems support healthier communities and emotional well-being. It was found that educational barriers such as lack of institutional support, insufficient educator training, and a lack of culturally relevant educational materials exist. The results suggest that incorporating ecological traditions in mental health education can lead to greater psychological awareness, decrease stigma, and promote holistic learning experiences that can connect emotional well-being with environmental sustainability.

### 3.4 Role of the elders and intergenerational knowledge transfer

In the study areas, community elders played a key role in preserving ecological knowledge and providing mental health support; interviews suggested that elders functioned as informal counselors, mediators, and emotional guides for younger generations, and participants stressed the significance of intergenerational relationships for maintaining community well-being. Elders shared ecological knowledge through storytelling, agricultural training, medicinal plant teaching, ceremonial involvement, and oral history. These interactions reinforced cultural identity and provided emotional security for youth.

Participants who had frequent contact with elders reported higher levels of social support and feelings of belonging, younger community members perceived elders as respected individuals who could provide guidance, conflict resolution, and emotional support, and the structured times for intergenerational interaction that arise out of community rituals and ecological activities increased the respect for cultural traditions and environmental stewardship, with youth who participated in ecological learning activities feeling more comfortable discussing mental health issues and seeking social support, and elders reporting a sense of purpose and emotional satisfaction from participating in educational programs.

**Figure 5:**
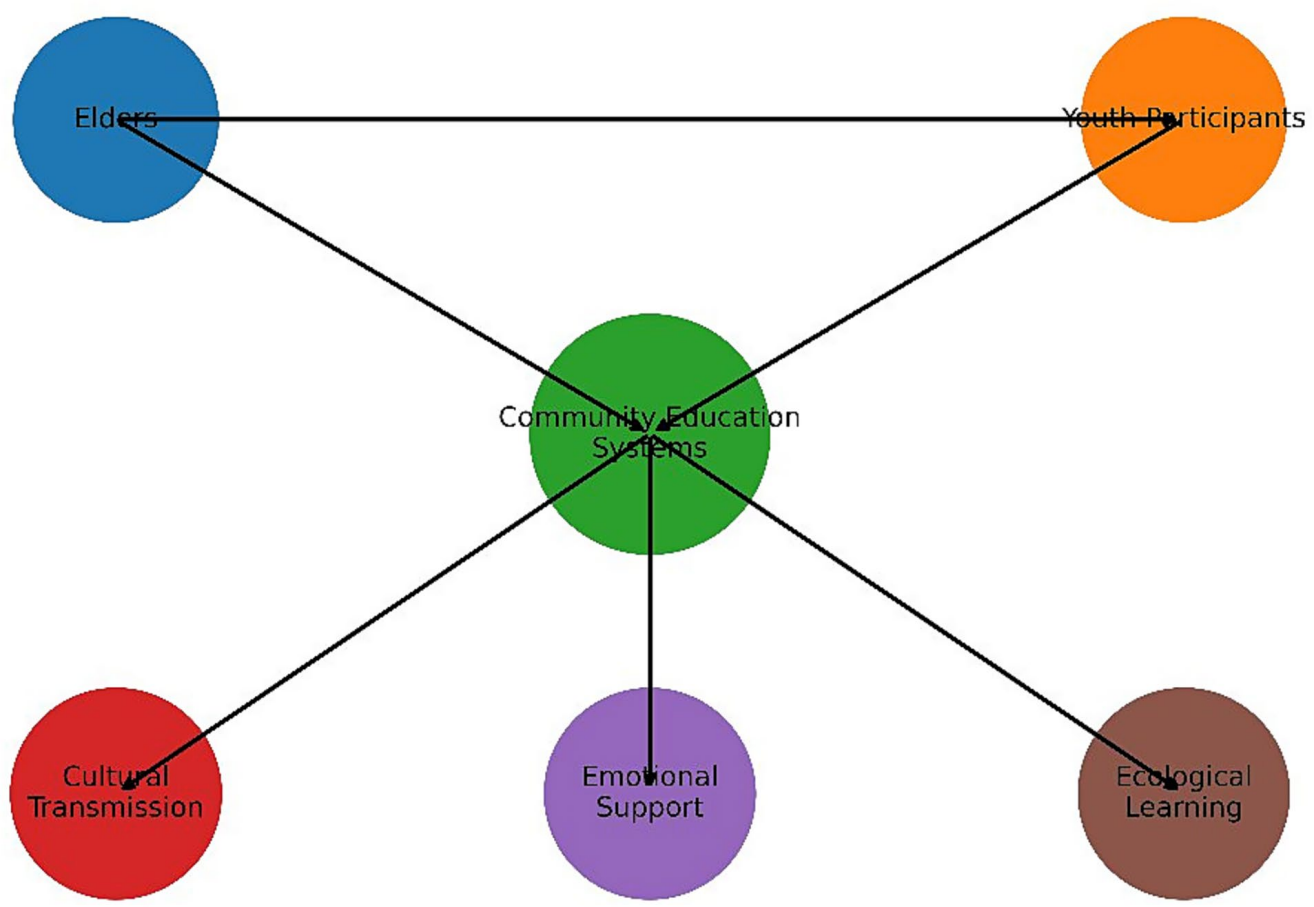
Intergenerational knowledge transfer and community support networks.

Figure 5 depicts the roles that elders and youth participants can play in facilitating cultural transmission, emotional support and ecological learning (e.g., migration, modernization, and formal educational systems may limit these traditional learning relationships) and the need for some communities to have structured programs to facilitate knowledge transfer between elders and youth. The findings emphasize the importance of the elders in supporting sustainable community well-being through cultural continuity, ecological education and emotional mentorship (Figure 5). Therefore, intergenerational learning initiatives may be important for both mental health promotion and the preservation of traditional ecological knowledge.

### 3.5 Environmental stewardship and sustainable community practices

The results showed that traditional ecological knowledge and sustainable environmental practices were closely linked in the participating communities: ecological traditions promoted conservation behaviors, responsible resource management, and collective environmental responsibility, which led to the adoption of sustainable agricultural techniques, water conservation, biodiversity protection, and seasonal land management practices, as well as environmental stewardship viewed as a cultural obligation and a social responsibility to future generations. Analysis of the survey data showed that communities with an active ecological education program had greater participation in environmental initiatives such as tree planting, waste reduction and ecosystem restoration activities, and that caring for the natural environment was directly linked to community health and psychological well-being.

Interview data showed that environmental degradation caused emotional distress and concern within communities, and loss of biodiversity, reduced access to traditional lands and climate-related disruptions were linked to anxiety, grief and cultural disconnection, making ecological restoration projects not only environmental interventions, but emotionally healing community activities. Community-based environmental programs also enhanced cooperation and social trust, and collective participation in farming, conservation work and cultural ceremonies fostered shared responsibility and reduced social isolation. Participants stressed that ecological sustainability and community mental health should not be considered in isolation. The challenges to sustainable ecological practices included economic pressures and external development projects, which some communities had difficulty reconciling with traditional conservation values.

**Figure 6:**
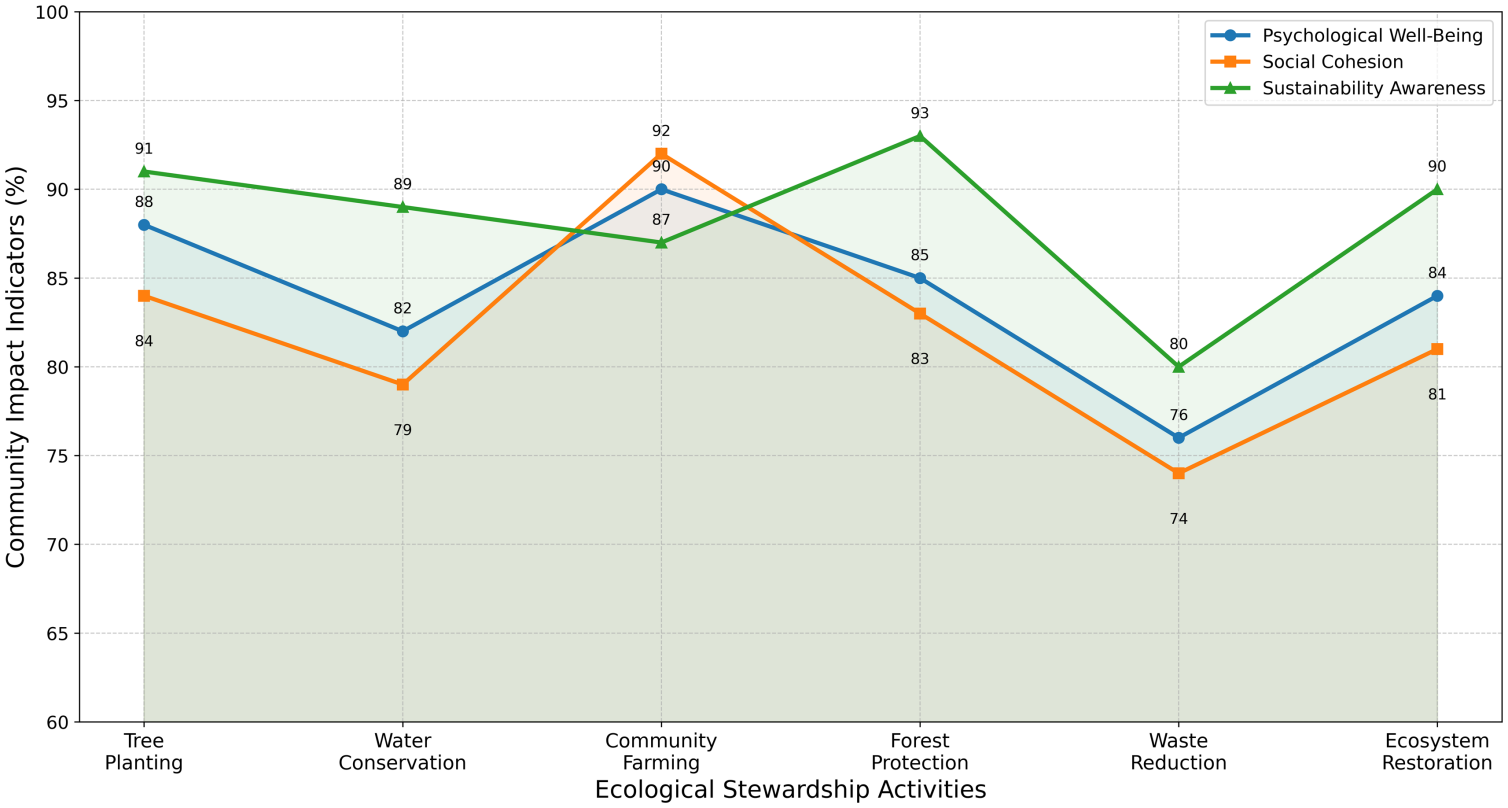
Sustainable environmental stewardship practices supporting community mental health.

Figure 6 presents the relationship between ecological stewardship activities and indicators of the psychological well-being, social cohesion and sustainability awareness within participating communities. These findings confirm that traditional ecological knowledge supports sustainable environmental stewardship while simultaneously promoting social cohesion, cultural continuity and emotional well-being (Figure 6).

### 3.6 Challenges in integrating traditional ecological knowledge into formal systems

Community members recognized many benefits of the traditional ecological knowledge, but institutional and social barriers to integrating TEK into formal mental health and educational systems were identified. The most common barrier was the dominant educational curriculum that emphasized scientific and technological subjects over indigenous knowledge systems, which limited time for TEK in the classroom and lacked policy support for their integration into educational programs, as teachers and community educators reported.

Healthcare practitioners noted that formal mental health systems often neglected cultural and environmental dimensions of well-being and that clinical approaches emphasized individual treatment rather than community relationships, ecological engagement or cultural healing practices, which they believed weakened the effectiveness of the mental health outreach efforts in culturally diverse communities.

Table 5 outlines key qualitative themes that emerged from interviews and focus group discussions regarding ecological traditions and their connection to mental health and community sustainability. Participation in ecological traditions also waned due to modernization and urban migration, as younger generations became more exposed to digital technologies and urban lifestyles, leading to less interaction with elders and natural environments, and some participants saw traditional practices as outdated or incompatible with contemporary education systems. Language loss was another major issue, as many ecological concepts and ceremonial meanings and cultural teachings were encoded within indigenous languages that are endangered, and participants said language decline poses a threat to both ecological knowledge preservation and cultural identity.

**Table 5:** Major qualitative themes emerging from participant narratives.

Major theme	Representative participant perspective	Community interpretation
Cultural identity	“Traditional practices help us remember who we are.”	TEK strengthens belonging and identity
Ecological healing	“Nature provides emotional peace and balance.”	Environmental interaction supports mental wellness
Intergenerational learning	“Elders teach us how to live responsibly.”	Knowledge transfer strengthens resilience
Community cohesion	“Working together reduces loneliness.”	Collective ecological activities improve social support
Sustainability awareness	“Protecting nature protects our future.”	Ecological responsibility promotes long-term well-being
Educational inclusion	“Children learn better through cultural experiences.”	TEK improves educational participation

**Figure 7:**
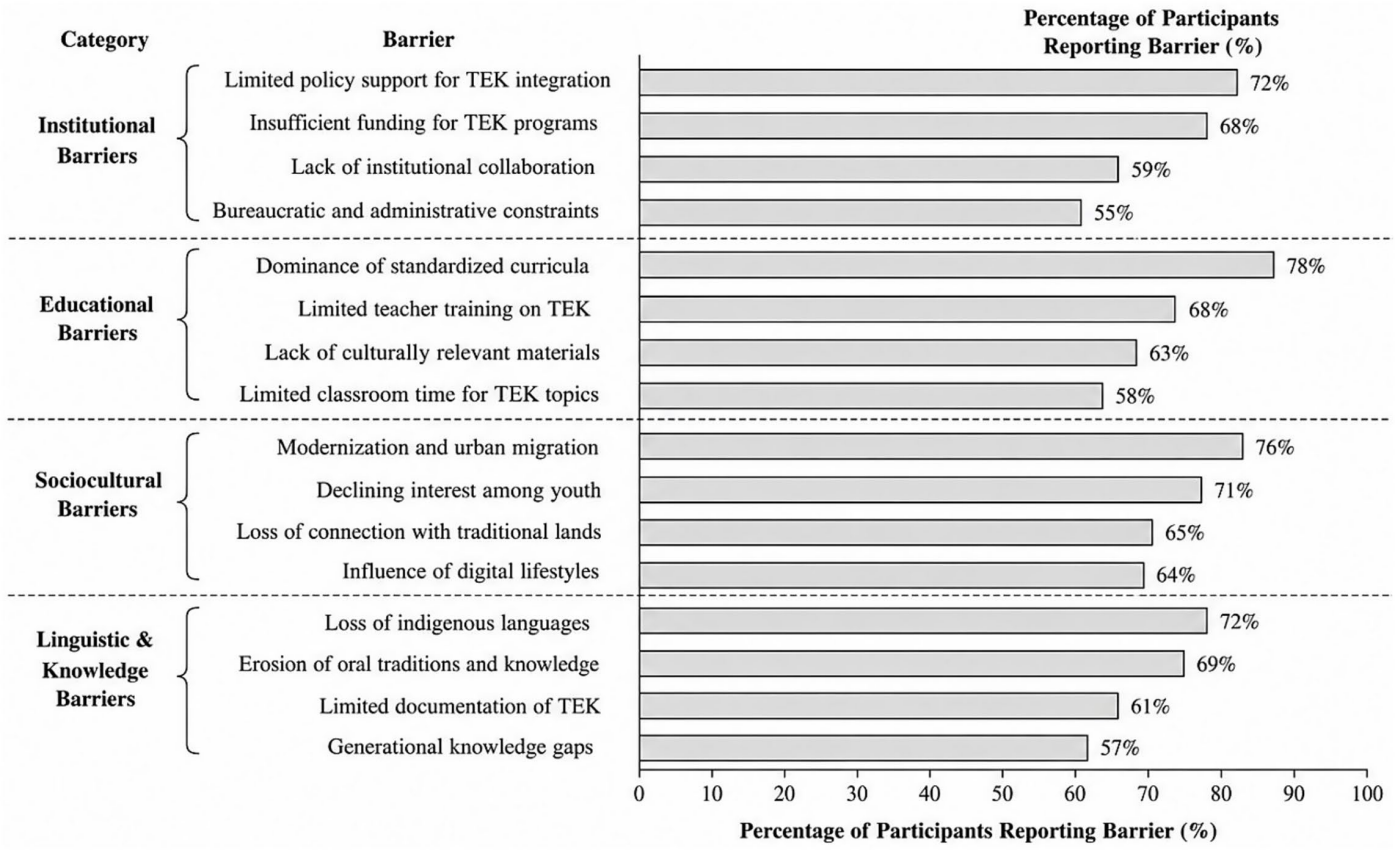
Institutional and sociocultural barriers affecting integration of traditional ecological knowledge.

Figure 7 illustrates the major institutional, educational, sociocultural and linguistic barriers limiting the integration of traditional ecological knowledge into the formal mental health and sustainability programs.

Despite these challenges, communities expressed interest in collaborative educational models that integrate scientific knowledge with traditional ecological perspectives (Table 6), and participants suggested greater policy support, culturally inclusive curricula, teacher training programs, and community partnerships to enhance integration efforts, which indicate the need to overcome institutional barriers to promote sustainable mental health education and preserve valuable ecological knowledge systems for future generations.

**Table 6:** Policy and educational recommendations for integrating traditional ecological knowledge into mental health systems.

Challenge identified	Recommended intervention	Key stakeholders	Expected impact
Decline in ecological knowledge	Community-based cultural education programs	Schools and community leaders	Improved cultural continuity
Limited TEK in curricula	Integration of indigenous perspectives in education	Educational policymakers	Inclusive learning systems
Mental health stigma	Culturally grounded awareness campaigns	Public health agencies	Increased mental health literacy
Weak intergenerational engagement	Elder–youth mentorship initiatives	Local organizations	Enhanced social cohesion
Environmental degradation	Community conservation projects	Environmental agencies	Sustainable ecosystem management
Lack of institutional support	Policy frameworks supporting TEK integration	Governments and NGOs	Long-term sustainability planning

## 4 Discussion

The present study examined the contribution of Traditional Ecological Knowledge (TEK) to mental health education and sustainable community well-being among indigenous and rural communities in Southwestern China, including Yi, Miao (Hmong), Dong, and Yao. The findings demonstrate that higher participation in TEK practices was significantly associated with improved emotional resilience, mental health awareness, social connectedness, cultural identity, and sustainability engagement. Statistical analyses showed strong positive associations among TEK participation, emotional resilience, mental health awareness, and sustainability engagement, and multiple regression analysis indicated that TEK participation was the most significant predictor of emotional resilience, which was corroborated by qualitative narratives describing how ecological traditions, intergenerational learning, and community participation contribute to psychological well-being.

The study also found a statistically significant relationship between participation in TEK and emotional resilience, with participants who reported high levels of participation in ecological traditions reporting higher resilience scores, lower stress levels, and stronger social support networks than participants reporting lower levels of ecological participation. The correlation analysis revealed a strong positive association between TEK participation and emotional resilience (r = 0.78, *p* < 0.01), while regression analysis confirmed that TEK participation was the strongest independent predictor of emotional resilience (*β* = 0.48, *p* < 0.001). These results align with findings in environmental psychology that meaningful interaction with nature helps regulate emotions, reduce stress, and restore psychological well-being (Leavell et al., 2019). Cultural activities that include the use of plants for medicine, community gardening, storytelling, and seasonal rituals may provide culturally meaningful avenues for coping and resilience. The results also align with culturally integrated models of mental health promotion, which conceptualize that psychological well-being is determined by cultural identity, community relationships, environmental connectedness, and collective meaning systems rather than just psychological processes alone (Naeim and Basharpoor, 2026; Mikaeili et al., 2025). The present findings extend these frameworks by providing empirical evidence that TEK functions as a culturally grounded psychosocial resource capable of strengthening resilience while simultaneously supporting environmental stewardship and cultural continuity.

Another significant finding relates to the relationship between TEK and cultural identity. Participants consistently explained that ecological traditions are significant contributors to feelings of belonging, meaning, and social cohesion, and communities that were more engaged in traditional ecological practices demonstrated higher levels of cultural identity and community connectedness. These findings are consistent with earlier research on cultural continuity acting as a buffer to psychological distress by increasing social support and reinforcing group values (Ianni et al., 2015), and with the associations between cultural identity, social connectedness, and resilience that are consistent with the Socio-Cognitive Integration Theory (SCIT), which posits that psychological well-being develops through the interaction between cultural values, social relationships, collective cognition, and environmental contexts (Narimani and Naeim, 2025).

The most prominent themes to emerge from the study was intergenerational learning, with elders playing key roles in the transmission of ecological knowledge, cultural values, and emotional coping strategies through means such as storytelling, ceremonial participation, medicinal plant education, and agricultural training (Hammood et al., 2025). Participants commonly described elders as confidants who would guide them through periods of social or personal strife, suggesting that multigenerational resilience theory is supported, whereby resilience is passed down through generations through cultural teachings, spirituality, collective memory, and social support systems (Hammood et al., 2025). The findings also showed that incorporating TEK into mental health education improves psychological awareness and educational engagement, with communities implementing culturally grounded educational activities reporting significantly higher awareness of stress management, emotional well-being, and community mental health resources (Datta, 2023). Of note, the study highlights that mental health education may be enhanced when psychological concepts are framed within culturally familiar ecological experiences and community traditions.

Environmental stewardship also emerged as an important aspect of community well-being; participants consistently associated environmental conservation with psychological health, cultural responsibility, and collective resilience, with communities actively engaged in conservation programs having greater sustainability awareness, social cooperation, and psychological well-being (Galvin, 2020). There was evidence that ecological restoration activities, tree planting programs, and conservation initiatives were often considered to be emotionally significant experiences that helped to build social cohesion and a sense of collective efficacy. However, a number of barriers to the integration of TEK into mental health practice were identified, including a lack of space for TEK in formal educational systems, a lack of integration of TEK into conventional mental health systems (which tend to focus on individualized treatment models that neglect cultural, environmental, and community aspects of well-being), and modernization, urban migration, lack of ecological participation by youth, and language loss, which are all consistent with studies indicating that socio-cultural erosion may also contribute to psychological distress, weakened cultural identity, and reduced social cohesion (Azar et al., 2024). The results thus indicate that the maintenance of indigenous languages and ecological traditions should be considered as much more than a cultural goal, but also a vital aspect of community mental health promotion.

The implications for this study for policy and practice include the following: indigenous ecological knowledge, environmental ethics, and land-based learning experiences should be integrated into culturally responsive curricula in educational systems; elders, community leaders, and traditional knowledge holders should be involved in educational planning and implementation of mental health promotion programs; and public health agencies should consider the interdependence of environmental sustainability, cultural continuity, and psychological well-being when designing community-based interventions. These emerging frameworks for mental health promotion are culturally responsive, preventive, and community-centered, and they integrate local knowledge systems into public health strategies (Mirzai et al., 2024; Naeim, 2024a; Naeim, 2024b; Naeim and Narimani, 2026).

The present findings empirically support these emerging frameworks and suggest the practical utility of TEK-informed mental health education. Some limitations must be acknowledged. The study is limited to the communities in Southern China, which may not fully capture the richness of indigenous ecological knowledge systems in various cultural contexts, and the cross-sectional design precludes causal interpretation of the observed relationships, so the findings should be interpreted as important associations rather than as direct causes. Longitudinal and comparative studies are recommended to investigate the long-term effects of TEK-based interventions on mental health outcomes and sustainable community development.

## 5 Conclusion

The results of this mixed-methods study indicate that TEK is a culturally and environmentally significant resource that can greatly benefit mental health education and sustainable community well-being. In the indigenous and rural communities of Southern China, a higher degree of participation in ecological traditions such as storytelling, medicinal plant use, community farming, land-based learning, environmental stewardship, and cultural ceremonies was correlated with higher levels of emotional resilience, social connectedness, mental health awareness, and sustainability engagement. As supported by quantitative findings showed a significant positive correlation between TEK participation, mental health awareness, and sustainability engagement. Multiple regression analysis also indicated that TEK participation was the most influential predictor of emotional resilience. It suggests that culturally embedded ecological practices may have significant psychological value. Qualitative findings further strengthened these results by showing how TEK participation enhances cultural identity, intergenerational relationships, emotional support, and collective responsibility for environmental conservation.

The study also underscores the importance of elders and traditional knowledge holders in maintaining cultural continuity, transmitting coping strategies, and providing community-based mental health learning, a finding that is consistent with culturally integrated mental health frameworks that place significant emphasis on community involvement, cultural identity, and communal resilience (Naeim and Basharpoor, 2026; Hammood et al., 2025). It respondents the identified barriers to TEK integration, including curricular constraints, modernization demands, diminishing use of indigenous languages, institutional apathy, and diminished opportunities for intergenerational knowledge transfer, all of which will necessitate culturally responsive educational policies, enhanced collaboration between educators and indigenous peoples, and mental health programs that recognize the interrelatedness of environmental, cultural, and psychological aspects of wellness.

The study contributes to growing body of the evidence supporting culturally grounded and sustainability-oriented approaches to the mental health promotion. Integrating TEK into the educational and community health initiatives offers the promising pathway for strengthening resilience, preserving cultural heritage, promoting environmental responsibility and advancing the holistic community development. Future longitudinal and comparative studies are recommended to further examine the long-term impacts of TEK-based mental health education across diverse sociocultural contexts.

## Data Availability

The original contributions presented in the study are included in the article/supplementary material, further inquiries can be directed to the corresponding author.
